# Genetic Variation in the Social Environment Contributes to Health and Disease

**DOI:** 10.1371/journal.pgen.1006498

**Published:** 2017-01-25

**Authors:** Amelie Baud, Megan K. Mulligan, Francesco Paolo Casale, Jesse F. Ingels, Casey J. Bohl, Jacques Callebert, Jean-Marie Launay, Jon Krohn, Andres Legarra, Robert W. Williams, Oliver Stegle

**Affiliations:** 1 European Molecular Biology Laboratory, European Bioinformatics Institute, Wellcome Genome Campus, Hinxton, United Kingdom; 2 Department of Genetics, Genomics and Informatics, University of Tennessee Health Science Center, Memphis, Tennessee, United States of America; 3 AP-HP, Hôpital Lariboisière, Department of Biochemistry, INSERM U942, Paris, France; 4 Wellcome Trust Centre for Human Genetics, Oxford, United Kingdom; 5 INRA, UMR 1388 GenPhySE, Castanet Tolosan, France; Stanford University, UNITED STATES

## Abstract

Assessing the impact of the social environment on health and disease is challenging. As social effects are in part determined by the genetic makeup of social partners, they can be studied from associations between genotypes of one individual and phenotype of another (social genetic effects, SGE, also called indirect genetic effects). For the first time we quantified the contribution of SGE to more than 100 organismal phenotypes and genome-wide gene expression measured in laboratory mice. We find that genetic variation in cage mates (i.e. SGE) contributes to variation in organismal and molecular measures related to anxiety, wound healing, immune function, and body weight. Social genetic effects explained up to 29% of phenotypic variance, and for several traits their contribution exceeded that of direct genetic effects (effects of an individual’s genotypes on its own phenotype). Importantly, we show that ignoring SGE can severely bias estimates of direct genetic effects (heritability). Thus SGE may be an important source of “missing heritability” in studies of complex traits in human populations. In summary, our study uncovers an important contribution of the social environment to phenotypic variation, sets the basis for using SGE to dissect social effects, and identifies an opportunity to improve studies of direct genetic effects.

## Author Summary

Daily interactions between individuals can influence their health both in positive and negative ways. Often the mechanisms mediating social effects are unknown, so current approaches to study social effects are limited to a few phenotypes for which the mediating mechanisms are known *a priori* or suspected. Here we propose to leverage the fact that most traits are genetically controlled to investigate the influence of the social environment. To do so, we study associations between genotypes of one individual and phenotype of another individual (social genetic effects, SGE, also called indirect genetic effects). Importantly, SGE can be studied even when the traits that mediate the influence of the social environment are not known. For the first time we quantified the contribution of SGE to more than 100 organismal phenotypes and genome-wide gene expression measured in laboratory mice. We find that genetic variation in cage mates (i.e. SGE) explains up to 29% of the variation in anxiety, wound healing, immune function, and body weight. Hence our study uncovers an unexpectedly large influence of the social environment. Additionally, we show that ignoring SGE can severely bias estimates of direct genetic effects (effects of an individual’s genotypes on its own phenotype), which has important implications for the study of the genetic basis of complex traits.

## Introduction

Social interactions contribute to health and disease (e.g. peer smoking increases one’s risk of taking up smoking). So far, quantifying social effects has required a clear hypothesis about the mechanisms mediating the influence of the social environment (in the example above peer smoking is the trait that mediates the social influence). For many phenotypes however, such hypotheses do not exist. Therefore we propose an alternative strategy to study social effects: we investigate effects on an individual's phenotype that arise from genotypes of social partners (social genetic effects, SGE, also called indirect genetic effects[1, 2]). SGE constitute the genetic basis of social effects and can be detected without prior knowledge of the phenotypes through which the social influence is exerted. SGE have been reported for interactions between mothers and offspring (maternal genotypes indirectly affect offspring phenotypes)[3–8] and more recently for interactions between adult individuals, in livestock and wild animals[2, 9–17] For example, growth rate in farm pigs has been found to be in part determined by the genetic makeup of the other pigs in the pen[2]. However, the extent to which SGE explain variation in biomedical traits is largely unknown.

If SGE do contribute to such traits, they are a promising approach to quantify effects of the social environment. Additionally, they provide an anchor to investigate causal paths and dissect the mechanisms underlying social effects. Finally, in studies of direct genetic effects carried out by the broad community, SGE may be used to account for social environmental effects.

Our study aimed at quantifying the contribution of SGE to multiple biomedical traits. We uncover unexpectedly large social genetic effects on multiple organismal and molecular phenotypes.

## Results

To investigate whether SGE explain variation in biomedical traits, we considered two experiments in laboratory mice involving complementary genetic designs, and assessed both organismal and gene expression traits.

### Experiment with two inbred strains

**Fig 1:**
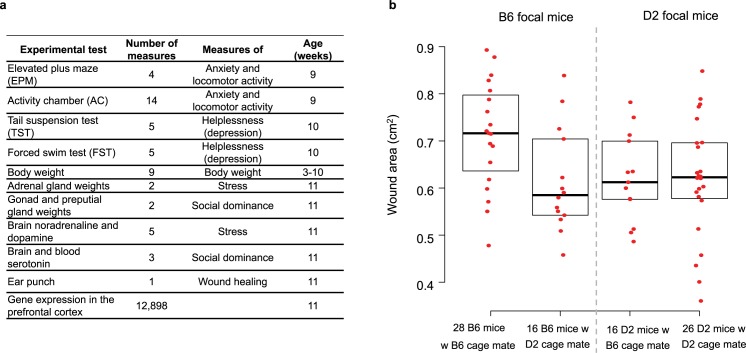
Experiment with two inbred strains. (A) Phenotypes collected in this experiment. Organismal phenotypes were measured on each of the 86 mice; after quality control, gene expression data was available for 79 mice (Methods). (B) Example phenotype (Wound area, from an ear punch, a measure of the rate wound healing) showing that phenotypic variation arises from variation in the strain of the focal (i.e. phenotyped) mouse (direct genetic effects, DGE), variation in the strain of its cage mate (social genetic effects, SGE), and an interaction between the two whereby the effect of the strain of the cage mate depends on the strain of the focal mouse. B6: C57BL/6J, D2: DBA/2J.

We first carried out an experiment with two inbred strains. We chose C57BL/6J (B6) and DBA/2J (D2), the progenitor strains of the largest mouse reference population, the BxD recombinant inbred panel[18]. We co-housed 86 mice at weaning as B6/B6, B6/D2 or D2/D2 pairs. After a period of six weeks during which the mice interacted undisturbed in their home cages, we collected 50 organismal phenotypes relevant to unconditioned anxiety, helplessness (a measure of depressed mood), general locomotor activity, stress, social dominance, wound healing and body weight (Fig 1A, S1 Table). We also profiled genome-wide gene expression in the prefrontal cortex (PFC). The PFC was selected because it is involved in coordinating behavioural responses based on sensorimotor information, motivation and affect, all of which may be affected by the social environment; as a result individual differences in PFC expression levels may reflect behavioural responses to the social environment [19–21].

In this design with two inbred strains there are three potential genetic sources of phenotypic variation: differences in the strain of the focal (i.e. phenotyped) mice (DGE), differences in the strain of their cage mates (SGE) and an interaction between the two, whereby the effect of the strain of the cage mate depends on the strain of the focal mouse (Fig 1B, S1 Fig and Methods). Variance partitioning and model selection (see Methods) provided evidence that interactions between DGE and SGE are common across traits (S2 Fig), with SGE typically affecting a specific trait in one strain (B6 or D2) but not the other (e.g. Fig 1B, S3 Fig). Thus, for each trait, we modeled the measurements collected in B6 mice and D2 focal mice separately (S1 Fig).

For eleven out of 50 organismal phenotypes we found significant SGE in either B6 or D2 mice (P < 0.05, FDR < 43%—see Methods; Table 1, S2 and S3 Tables). Strain-specific differences existed as different measures were affected by SGE in B6 and D2 mice. Of these eleven phenotypes, two were measures of stress and six were measures of anxiety, providing evidence that variation in the genetic makeup of cage mates causes variation in stress-related phenotypes. The direction of effect was consistent across the six measures of anxiety (S3 Fig). We also detected strong SGE on the rate of wound healing (measured from an ear punch, P = 6.6 10^−3^, Q = 0.36), showing that SGE are not limited to behaviors. (Table 1). Importantly, SGE explained a considerable proportion of phenotypic variance (up to 18%), showing that social effects of genetic origin are important contributors to phenotypic variation in these two strains.

**Table 1:** Organismal phenotypes significantly affected by SGE in the experiment with inbred strains (P < 0.05). B6 and D2 focal mice were analysed separately. SGE Q value: see Methods. SGE variance (%): Percentage of phenotypic variance explained by SGE +/- standard error (see Methods). * the expression in the PFC and in B6 focal mice of genes associated with the regulation of dopamine metabolism is also affected by SGE (Table 2).

Focal mice	Phenotype	Measure of	SGE P value	SGE Q value	SGE variance (%)
B6	Wound area	Wound healing	6.6 10^−3^	0.36	18 +/- 12
B6	Midbrain concentration of noradrenaline	Stress	7.2 10^−3^	0.36	15 +/- 10
B6	Midbrain concentration of dopamine*	Stress	2.6 10^−2^	0.38	10 +/- 9
D2	Time spent not moving in center first min (AC)	Anxiety	1.1 10^−2^	0.38	15 +/- 11
D2	Total arm entries (EPM)	Locomotor activity	1.6 10^−2^	0.38	12 +/- 10
D2	Ratio time spent not moving periphery/center min 2 to 5 (AC)	Anxiety	2.6 10^−2^	0.38	14 +/- 11
D2	Ratio time spent not moving periphery/center min 6 to 30 (AC)	Anxiety	3.2 10^−2^	0.38	11 +/- 9
D2	Number immobility bouts first 2 min (FST)	Helplessness	3.4 10^−2^	0.38	8 +/- 8
D2	Ratio time spent periphery/center min 2 to 5 (AC)	Anxiety	3.5 10^−2^	0.38	12 +/- 10
D2	Ratio ambulatory time periphery/center min 6 to 30 (AC)	Anxiety	3.8 10^−2^	0.38	9 +/- 8
D2	Proportion of entries in open arms (EPM)	Anxiety	4.8 10^−2^	0.43	10 +/- 9

**Table 2:** Gene set enrichment analysis based on the contribution of SGE to gene expression in the prefrontal cortex (PFC) in the experiment with inbred strains. The analysis was carried out in B6 and D2 focal mice separately. Enrichment was calculated based on the contribution of SGE to gene expression variance (see Methods). The 5 most significant GO terms for each of strain are shown. * dopamine measured by HPLC in the midbrain is also strongly affected by SGE (Table 1).

Focal mice	GO term ID	GO term	# annotated genes	P value	Q value
B6	GO:0042053	regulation of dopamine metabolic process*	12	7.0 10^−4^	1
B6	GO:0045216	cell-cell junction organization	127	2.8 10^−3^	1
B6	GO:0072376	protein activation cascade	20	2.8 10^−3^	1
B6	GO:1902017	regulation of cilium assembly	15	3.3 10^−3^	1
B6	GO:0042987	amyloid precursor protein catabolic process	19	3.4 10^−3^	1
D2	GO:0007229	integrin-mediated signaling pathway	57	2.5 10^−6^	0.024
D2	GO:0043269	regulation of ion transport	431	1.7 10^−4^	0.81
D2	GO:0030001	metal ion transport	543	1.4 10^−3^	1
D2	GO:1900006	positive regulation of dendrite development	59	1.5 10^−3^	1
D2	GO:0008542	visual learning	43	1.9 10^−3^	1

We also found evidence that SGE affect gene expression in the PFC. A gene set enrichment analysis based on the contribution of SGE to gene expression levels (see Methods) revealed “integrin-mediated signaling pathway”(P = 2.5 10^−6^, Q = 0.024) and “regulation of dopamine metabolic process” (P = 7.0 10^−4^, Q = 1) as the most significant Gene Ontology (GO) terms in D2 and B6 mice respectively (Table 2). The latter enrichment, although statistically less robust, is strikingly consistent with our finding that SGE affect dopamine levels measured by HPLC in B6 mice (Table 1). Our results therefore converge to show that variation in the genetic makeup of cage mates causes variation in behavioral, biochemical, and gene expression traits relevant to stress. Although the large number of tests limits the statistical power to detect SGE on individual genes (12,898 genes tested), we identified three genes with significant effects (*Srsf2*, *Phlpp2*, and *Ppid;* FDR < 33%; S4 Table).

### Reanalysis of a large outbred mice dataset

We next investigated SGE in a dataset from outbred (Heterogeneous Stock) mice[22–24]. This design better represents traditional mouse housing conditions (groups of four, five and six mice mostly rather than pairs; S4 Fig) and genetic variation in natural populations (high genetic diversity and genetically unique individuals rather than two inbred strains). The dataset comprises more than 100 organismal phenotypes measured in 2,448 mice (S5 Table), and gene expression in hippocampus for a subset of 457 mice. To accommodate the genetic design of this study, we fitted random effects models with variance components for DGE, SGE and their covariance. Our models are inspired from models published in the literature[2, 25] yet differ in some important ways, which we explain and justify in S1 Note. Because genome-wide genotypes were not available for a subset of mice (526 out of 2,448), we used pedigree information to estimate pairwise genetic covariance for the mice with no genotype information (see Methods). We found that the results obtained using both pedigree and genotype data for estimation of genetic similarity were in agreement with those obtained using genotype data only from the subset of mice that were in cages where all mice had been genotyped (S5 Fig). All models were fitted using LIMIX[26, 27].

Simulations showed that DGE and SGE were unbiased (S6 Fig and S7 Fig). Of the 117 organismal phenotypes available in this dataset, 43 were significantly affected by SGE (P < 0.05, FDR < 5.7%; Table 3, S5 Table). SGE explained up to 29% of phenotypic variation and an average of 8.9% across the 43 significantly affected traits. Importantly, the estimated contribution of SGE was greater than that of DGE for 8 of the 43 traits.

**Table 3:** Organismal phenotypes significantly affected by SGE in the outbred mice dataset (P < 0.05). Q value: see Methods. STE: standard error. Bold text highlights eight phenotypes for which the contribution of SGE is greater than that of DGE (contribution of SGE–STE > contribution of DGE + STE).

*Measure*	*Measure of*	*SGE P value*	*SGE Q value*	*SGE variance (%)*	*DGE variance (%)*
*CD4 fluorescence intensity*	*Lymphocyte function*	*<1.00 10^-16^*	*<1.00 10^-16^*	***29 +/- 5***	*21 +/- 2*
*Glycemia before glucose injection*	*Basal glycemia*	*1*.*30 10^-10^*	*3*.*20 10^-09^*	*11 +/- 4*	*11 +/- 2*
*Size of CD4+ cells (forward scatter)*	*Lymphocyte function*	*2*.*50 10^-08^*	*4*.*40 10^-07^*	***27 +/- 6***	*5 +/- 1*
*Size of B cells (forward scatter)*	*Lymphocyte function*	*4*.*00 10^-08^*	*5*.*20 10^-07^*	***25 +/- 6***	*5 +/- 1*
*Size of CD8+ cells (forward scatter)*	*Lymphocyte function*	*8*.*00 10^-08^*	*8*.*20 10^-07^*	***27 +/- 6***	*4 +/- 1*
*Chloride*	*Blood biochemistry*	*7*.*70 10^-07^*	*6*.*60 10^-06^*	***17 +/- 5***	*4 +/- 2*
*Calcium*	*Blood biochemistry*	*9*.*90 10^-07^*	*7*.*30 10^-06^*	***18 +/- 5***	*5 +/- 2*
*Absolute number of lymphocytes*	*Lymphocyte function*	*3*.*60 10^-06^*	*2*.*30 10^-05^*	*7 +/- 4*	*9 +/- 3*
*Weight at 6 weeks of age*	*Body weight*	*5*.*30 10^-06^*	*3*.*00 10^-05^*	*7 +/- 4*	*11 +/- 2*
*Sodium*	*Blood biochemistry*	*5*.*90 10^-06^*	*3*.*00 10^-05^*	***17 +/- 5***	*2 +/- 2*
*Alanine transaminase*	*Liver function*	*1*.*00 10^-05^*	*4*.*70 10^-05^*	***11 +/- 4***	*2 +/- 2*
*White blood cells*	*Lymphocyte function*	*3*.*50 10^-05^*	*0*.*00014*	*6 +/- 4*	*7 +/- 3*
*Mean startle (following bang only) before conditioning with foot shock*	*Unconditioned anxiety*	*0*.*00011*	*4*.*00 10^-04^*	*15 +/- 4*	*24 +/- 4*
*Weight before food hyponeophagia test (7 weeks)*	*Body weight*	*0*.*00015*	*0*.*00052*	*12 +/- 4*	*6 +/- 2*
*Glycemia 15 minutes after glucose injection*	*Glucose tolerance*	*0*.*00035*	*0*.*0011*	*9 +/- 4*	*9 +/- 3*
*High density lipoprotein*	*Blood biochemistry*	*0*.*00046*	*0*.*0014*	*8 +/- 4*	*35 +/- 3*
*Proportion of CD3+ T-cells that are CD4+*	*Lymphocyte function*	*0*.*00049*	*0*.*0014*	*7 +/- 5*	*9 +/- 3*
*Red blood cell distribution width*	*Red blood cell function*	*0*.*00053*	*0*.*0014*	*6 +/- 4*	*28 +/- 4*
*Weight after food hyponeophagia test*	*Body weight*	*0*.*00093*	*0*.*0024*	*10 +/- 4*	*7 +/- 2*
*Percentage of B220+ cells*	*Lymphocyte function*	*0*.*0011*	*0*.*0027*	*10 +/- 5*	*32 +/- 4*
*Wound (ear hole) area*	*Wound healing*	*0*.*0016*	*0*.*0037*	*6 +/- 3*	*32 +/- 3*
*Expiratory time (after metacholine)*	*Lung function*	*0*.*0018*	*0*.*0039*	*3 +/- 3*	*11 +/- 3*
*Enhanced pause (before metacholine)*	*Lung function*	*0*.*0019*	*0*.*0041*	*10 +/- 4*	*9 +/- 3*
*Distance traveled in the center of the EPM*	*Unconditioned anxiety*	*0*.*0021*	*0*.*0043*	*4 +/- 3*	*27 +/- 4*
*Mean startle (following tone + bang) before conditioning with foot shock*	*Unconditioned anxiety*	*0*.*0032*	*0*.*0063*	*9 +/- 4*	*25 +/- 4*
*Percentage of CD8+ cells*	*Lymphocyte function*	*0*.*0039*	*0*.*0073*	*4 +/- 3*	*48 +/- 4*
*Distance traveled in the closed arms of the EPM*	*Locomotor activity*	*0*.*0042*	*0*.*0077*	*2 +/- 3*	*18 +/- 3*
*Distance traveled in the open arms of the EPM*	*Unconditioned anxiety*	*0*.*0057*	*0*.*01*	*2 +/- 3*	*19 +/- 3*
*Number of entries into the open arms of the EPM*	*Unconditioned anxiety*	*0*.*0063*	*0*.*011*	*2 +/- 3*	*19 +/- 3*
*Albumin*	*Blood biochemistry*	*0*.*0066*	*0*.*011*	*5 +/- 4*	*4 +/- 2*
*Number of KI67+ cells*	*Adult neurogenesis*	*0*.*01*	*0*.*017*	*4 +/- 6*	*38 +/- 7*
*Measured mean cell hemoglobin concentration*	*Red blood cell function*	*0*.*012*	*0*.*019*	*7 +/- 4*	*15 +/- 3*
*Beam breaks from ambulation in home cage during last 5 minutes*	*Locomotor activity*	*0*.*013*	*0*.*02*	*2 +/- 3*	*11 +/- 3*
*Faecal steroids in males and females*	*Stress*	*0*.*015*	*0*.*022*	*6 +/- 5*	*8 +/- 4*
*Time spent in the open arms of the EPM*	*Unconditioned anxiety*	*0*.*015*	*0*.*022*	*1 +/- 3*	*17 +/- 3*
*Aspartate transaminase*	*Blood biochemistry*	*0*.*023*	*0*.*032*	*4 +/- 4*	*2 +/- 2*
*Alkaline phosphatase*	*Blood biochemistry*	*0*.*026*	*0*.*036*	*1 +/- 3*	*44 +/- 3*
*Creatinine*	*Blood biochemistry*	*0*.*027*	*0*.*036*	*5 +/- 5*	*2 +/- 3*
*Glucose*	*Basal glycemia*	*0*.*032*	*0*.*041*	*11 +/- 4*	*9 +/- 3*
*Time freezing during cue*	*Conditioned anxiety*	*0*.*034*	*0*.*042*	*7 +/- 4*	*15 +/- 4*
*Percentage of CD3+ cells*	*Lymphocyte function*	*0*.*034*	*0*.*042*	*5 +/- 4*	*31 +/- 4*
*Respiratory rate (after metacholine)*	*Lung function*	*0*.*04*	*0*.*048*	*1 +/- 3*	*17 +/- 4*
*Expiratory time (before metacholine)*	*Lung function*	*0*.*049*	*0*.*057*	*2 +/- 3*	*15 +/- 3*

Among the organismal phenotypes most significantly and strongly affected by SGE in this dataset were measures of lymphocyte activation (in particular size and number of CD4+ T cells and B cells, collected by fluorescence-activated cell sorting and full blood count, Table 3). In contrast, measures related to other leucocytes (neutrophils, basophils and monocytes) and natural killer T cells. Altogether, these results indicate that genotypes of cage mates influence humoral immunity, although this is unlikely that this represents spread of a disease as the mice were kept in a clean mouse facility.

In addition, rate of wound healing, the measure most significantly and strongly associated with SGE in the experiment with two inbred strains, was also significantly affected by SGE in the outbred dataset (P = 1.6 10^−3^, Q = 3.7 10^−3^).

Three measures of body weight (collected on weeks 6 and 7) also figured among the traits significantly affected by SGE. Other measures of body weight, collected at weeks 9 and 10, showed no SGE (S5 Table). The two sets of measures likely reflect a different phenotype as the normal physiology of the mice was disrupted between weeks 7 and 9 by aggressive phenotyping (tests of conditioned anxiety involving foot shocks, airway sensitization by allergen, intraperitoneal injection of glucose). Another potential but unlikely explanation for the higher contribution of SGE on earlier body weight measures is a sharp decrease in social effects between weeks 7 and 9.

Finally, a subset of measures of anxiety, blood biochemistry, and lung function were also affected by the genotypes of cage mates.

In contrast, there was no statistical evidence for SGE on gene expression levels in the hippocampus (smallest nominal P value 4.1 10^−5^ corresponding to a Q value of 64%, S6 Table). This is most likely the result of a much smaller sample size (457 or 5% of mice) for expression traits, and is consistent with published power analyses[28].

**Fig 2:**
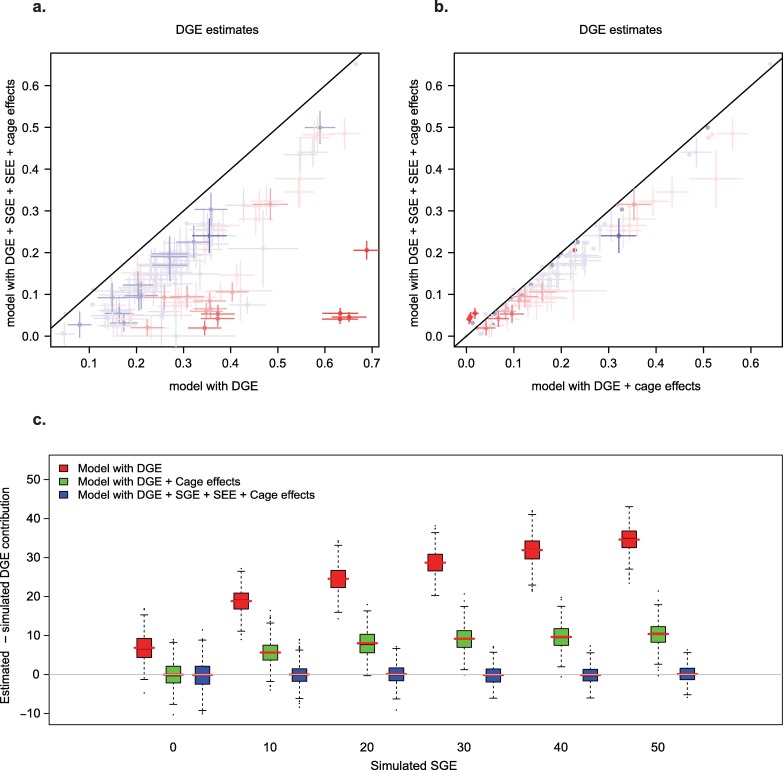
Ignoring SGE leads to biased estimates of DGE (heritability) in the outbred mice dataset. (A,B) DGE estimates for all organismal phenotypes. The colour of the dots indicates the sign of the covariance $\sigma_{A_{DS}}$ between DGE and SGE (blue: negative; red: positive; see Methods); the intensity of the colour indicates the magnitude of SGE. (A) Comparison of DGE estimates from model with DGE only (x-axis) and full model with DGE, SGE, (y-axis). (B) Comparison of DGE estimates from model with DGE and cage effects (x-axis) and full model (y-axis). (C) Difference between estimated and simulated DGE. The contribution of SGE to simulated phenotypes varied and is shown on the x-axis. See Methods for values given to other parameters. Simulations were analyzed with three different models: DGE only (red), DGE + cage effects (green), DGE + SGE + cage effects + social environmental effects (blue).

We next explored whether studies focused on DGE can safely ignore SGE, focusing on the estimation of the collective effect of additive DGE on phenotypic variation (narrow-sense heritability). In the outbred dataset, cage mates are genetically more similar to each other than average (S8 Fig). As a result, DGE and SGE are correlated (an example of gene-environment correlation). Thus, we hypothesized that failing to account for SGE would bias estimates of DGE. We also hypothesized that fitting cage effects might be sufficient to eliminate this bias. To investigate both hypotheses, we compared DGE estimates obtained using a linear mixed model for DGE that does not account for cage effects nor SGE, a model that accounts for DGE and cage effects, and one that accounts for DGE, SGE, corresponding social environmental effects, and cage effects (“full model”). Models that did not account for SGE led to substantially larger DGE estimates, and estimates from the model with DGE and cage effects were intermediate between those from the model with DGE only and those from the full model. In simulated traits based on the real genotypes and generated from DGE, SGE and cage effects (see Methods), we found that models that did not account for SGE yielded inflated DGE estimates, whereas joint modeling of DGE, SGE and cage effects resulted in unbiased estimates (Fig 2C and S6 Fig). Importantly, fitting cage effects but no SGE did not eliminate the bias. The simulation results strongly suggest that, in the real data, the estimates obtained from the full model are most accurate, and that models that ignore SGE overestimate heritability. This problem is particularly acute when direct and social random genetic effects are positively correlated (i.e. $\sigma_{A_{DS}}>0$, see Methods; Fig 2A and 2B). The problem we highlight here is general and likely to affect other studies in which social partners are related, including twin and family studies used to estimate heritability in humans (see Discussion).

## Discussion

Using two complementary genetic designs–one using two mouse inbred strains and one using outbred mice—we estimated the contribution of social genetic effects to a variety of organismal phenotypes and gene expression traits. The experiment with two inbred strains was designed to investigate SGE and focused on behaviours (anxiety and helplessness), as there is strong evidence that behaviours are socially affected[29–33]. To test whether SGE can be detected in outbred populations and survey a broader range of phenotypes, we re-analysed a large dataset from outbred mice and quantified the contribution of SGE to more than 100 phenotypes. The design of our study raises important questions: are positive results (i.e. evidence of SGE) in the experiment with two inbred strains expected to replicate in the outbred dataset? Are some phenotypes expected to be affected by SGE and some not? We now discuss these points.

Some phenotypes were measured in both experiments with similar protocols. Wound area, the measure of wound healing, was collected in both experiments using the exact same protocol. It is significantly affected by SGE in both experiments. The protocols for measuring body and adrenal gland weight are fairly simple thus reducing technical variation between experiments, and body weight was measured at about the same time point (around 50 days of age) in both experiments. There was strong evidence for SGE on body weight in the outbred dataset but no evidence in the experiment with two inbred strains. No SGE on adrenal gland weight were detected in either dataset. Finally, a partially overlapping set of measures of unconditioned anxiety was significantly affected by SGE in both experiments. While reviewing results from the two experiments in parallel is informative, positive results in one experiment are not strictly expected to replicate in the other. Indeed, although the variants that give rise to SGE in the experiment with two inbred strains also segregate in the outbred population (the two strains used in our experiment were among the eight founders of the outbred population), they have recombined with many additional variants from the six other founders. Moreover, the housing conditions were very different in the experiment with inbred strains and outbred experiment (group size of 2 vs. 2 to 7 respectively, and unfamiliar mice vs. familiar mice housed together). Therefore, one should not expect the overall contribution of SGE be the same in the two experiments. Rather, combining the two experiments provides a first hint at the generalizability of our results. Published studies of SGE provide additional information on this matter, and suggest that SGE may contribute to variation in body weight across species[2].

That social genetic effects contribute to variation in anxiety probably does not come as a surprise but their contribution to wound healing maybe more so. This result is however supported by significant p-values in both experiments of our study and large effect sizes (18 and 6%). When interpreting this result, it is important to bear in mind that social effects on wound healing can (and will, necessarily) be mediated by traits of cage mates that are different from wound healing. For example, social effects on wound healing could be mediated by social grooming, which could either mechanically disrupt the healing process or chemically enhance it[34]. Any traits of cage mates that may induce a systemic stress response in the focal animal could also mediate social effects on wound healing[35, 36]. Thus, social effects on wound healing are not unlikely, and, similarly, social effects may affect any phenotype (e.g. by through the induction of a systemic stress response,).

Because any phenotype may *a priori* be affected by social effects and the mechanisms at play are rarely known, SGE offer an attractive alternative to investigate social effects. First, as we have shown, they can be used to quantify social effects, effectively providing a lower bound estimate of social effects (as only the genetic component is captured). Second, SGE can be used to test whether a particular trait of social partners has an effect on a phenotype of interest. Establishing a causal relationship between two phenotypes is always difficult because of the risk of reverse causation and independent action of hidden confounders on both traits; SGE provide an anchor to test causality.

Independent of their relevance for studying social effects, we show that ignoring SGE can lead to biased estimates of heritability (i.e. the collective effect of DGE). In our study (outbred dataset), DGE and SGE are correlated by design (mice that share a cage are more genetically similar than average), and we show that this correlation leads to biased estimates of heritability if unaccounted for. Fitting cage effects, which has the primary goal of accounting for environmental effects shared by cage mates (e.g. noise levels), does not eliminate the bias. Our results are of interest to the broad genetics community as DGE and SGE are correlated in most if not all experimental designs traditionally used to estimate heritability in humans and model organisms, and SGE may thus have caused widespread bias. For example, in twin designs, MZ twins not only share 100% of their genotypes but they also share 100% of the genotypes of their sibling; DZ twins in comparison share both 50% of their genotypes and 50% of the genotypes of their sibling. Thus, SGE can contribute to increased concordance between MZ twins compared to DZ twins. If SGE are not modelled, heritability may be overestimated and appear “missing” when compared to genome-wide association results obtained from unrelated individuals[37]. Note that when the covariance $\sigma_{A_{DS}}$ between direct and social random genetic effects is negative (competition effects), ignoring SGE may lead to underestimating heritability. SGE in humans were considered once before (“sibling effects” [38]) but were never, to the best of our knowledge, modelled in heritability studies. Because we found that fitting cage effects was not sufficient to eliminate the bias due to SGE, we suspect that accounting for a “common environment” shared by family members, as is commonly done in human studies[39–41], will not eliminate SGE-induced bias.

It is not the first time that unaccounted for gene-environment correlations are put forward as potential causes of bias (e.g. Conley *et al*. investigated the correlation between genetics and urban setting [42]). However, the impact of the correlation between DGE and SGE is likely to be particularly severe as we have shown that SGE affect a wide range of phenotypes and DGE and SGE are correlated in most experimental designs used to estimate heritability.

Our study sheds light on an important component of the genetic architecture of complex traits, one that lies outside the individual, in social partners. Social genetic effects have already been shown to play an important role in artificial selection of livestock[43] and have important evolutionary consequences[44, 45]. Our results provide evidence that SGE are also an important component of health and disease.

## Methods

### Experiment with inbred strains

#### Animals and experimental design

Forty-five C57BL/6J (B6) and 45 DBA/2J (D2) about 3-weeks-old (±3 days) were shipped from The Jackson Laboratory. Mice were weaned on the day they were shipped and littermates were shipped together so as to minimise novel social interactions prior to experiments. Three D2 mice died during shipment and to maintain balance one B6 mouse was excluded. On arrival at UTHSC, mice were transferred to standard (75 square inches) mouse cages and allowed to recover overnight with their original littermates. Ears were punched for identification, and mice were transferred to new cages. Mice were co-housed in pairs to make up sixteen B6/D2 (BD) cages, 14 B6/B6 (BB) cages, and 13 D2/D2 (DD) cages assembled using mice from 15 B6 and 15 D2 litters (3 mice per litter originally). Littermates were never paired in BB and DD cages so cage mates were always unfamiliar with each other at the beginning of the experiment. Littermates were distributed across the three groups (BB, BD, and DD), and the cages ordered as successive BB, BD, and DD triplets on the shelves. Mice were housed in standard mouse cages with corncob bedding and a cardboard shelter on a fixed 12:12 hour light: dark cycle with *ad libitum* access to food and water. Phenotypes were collected after six weeks of co-housing.

#### Phenotyping

All protocols were approved by the UTHSC Institutional Animal Care and Use Committee. Mice were phenotyped between 7am and 2pm, in a random order different for each test. The order in which tests were performed, as well as the measures collected in each test, are summarised in S1 Table.

Elevated plus maze: We used an elevated plus maze (Columbus Instruments) with a surface ~30 cm above the floor that contained four 51-cm long, 11.5 cm-wide arms arranged at right angles. Closed arms had opaque walls 30 cm high, extending the length of the arm. Each mouse was placed in the center of the maze facing an open arm and allowed 5 minutes to explore the maze. Each cage mate was tested in turn and precaution was taken so that cage mates did not interact just after the first cage mate had finished the test and before the second one was taken to the maze.

Activity chamber: We used six 17" X 17" Plexiglas activity chambers (Med Associates Inc., ENV-510) with photobeams around the edge to measure movement. The beams were interfaced to a computer to record movement. Each activity chamber was located into a closed section of a cupboard, which had white light on. Each mouse was placed in the centre of the chamber and given 30 minutes to explore the activity chamber. Cage mates were tested at the same time.

Tail suspension test: Adhesive tape was wrapped around each tail three quarters of the distance from the base, and used to attach a suspension hook. Animals were suspended for six minutes and video recorded from the side. Cage mates were tested at the same time.

Forced swim test: Mice were placed in six cylinders (height: 20 cm, diameter: 15 cm) with water at about 25°C for 6 minutes and their behaviour recorded from the side. Upon completion mice were dried. Cage mates were tested at the same time.

Body weight: Mice were weighed on the day after arrival at UTHSC and every week thereafter.

Sacrifice, tissue collection, and gland weights: Mice were euthanized by injection of 250 mg/kg Avertin (20 mg/ml; tribromoethanol) followed by cardiac puncture and exsanguination under deep anaesthesia. The blood was collected in lithium heparin tubes, and an aliquot was taken in another lithium heparin tube for serotonin quantification. The aliquot was immediately snap frozen in liquid nitrogen and then stored at -80C.

The brain was dissected and placed in an ice-cold coronal mouse brain matrix. A slab containing the prefrontal cortex (PFC) was removed, and the PFC taken, snap frozen and stored at -80°C. The part of the brain caudal to the amygdala was dissected, snap frozen and stored at -80°C. Then the midbrain was dissected by letting the brain soften on ice and removing the cerebellum. The midbrain was stored at -80°C and shipped on dry ice for analysis by high-pressure liquid chromatography.

Both preputial glands were dissected and weighed together, as were both gonads. Right and left adrenal glands were weighed separately.

High-pressure liquid chromatography: Brain samples were weighed frozen. They were then crushed in 1,5 ml of 10^-3^M HCl containing sodium metabisulfite and ascorbic acid (antioxidants), and centrifuged at 22,000 g for 20min at 5°C. The supernatants were collected and filtered through a10-kDa membrane (Nanosep, Pall) by centrifugation at 7,000 g. Then, a 20 μl aliquot of sample was analyzed for serotonin by fluorometric detection[46]. The amounts of catecholamines (dopamine and noradrenaline) and their metabolites (DOPAC, HVA, VMA) as well as the serotonin metabolite 5HIAA were measured by electrochemical detection on a serial array of coulometric flow-through graphite electrodes (CoulArray, ESA)[47]. The analysis, data reduction and peak identification were fully automated.

Ten μl of blood were used for serotonin quantification. Serotonin was extracted by adding 400 μl of 10^−3^ M HCl, followed by 2 centrifuge runs including one on Nanosep 10 kD. Quantification was carried out as for brain serotonin.

Size of ear hole: At sacrifice the ear that was clipped on arrival was cut and placed in formalin. It was then mounted on a glass slide, scanned, and the resulting image analysed with ImageJ. The scale was set once using the size of the slide. The hole was delimited using the freeform drawing tool.

#### Pre-processing of organismal phenotypes

All collected phenotypes were quantitative. Individual phenotypes were normalized using the parametric Box-Cox power transformation[48], using R’s MASS package[49] and a model where strain of the animal, strain of the cage-mate, and an interaction between the two were included as fixed effects to inform the Box-Cox fit. Note that this approach does not regress out these genetic effects.

On the Box-Cox transformed data for each phenotype, we tested for association with experimental covariates and regressed out significantly associated covariates that were unrelated to the measure of interest. The covariates that were accounted for are reported in S1 Table. Significance of SGE calculated by including covariates in the models rather than regressing them out are reported in S3 Table.

#### RNA extraction and sequencing

Total RNA was extracted using a Qiagen Qiacube and RNeasy mini kit according to the manufacturers protocols and was loaded on a 96-well plate, with samples randomly allocated to wells, for shipment to the sequencing centre. The quantity and quality of the samples was assessed using Agilent high sensitivity R6K screen tape. One sample (B42) with high degradation was excluded. Two samples (B39, D30) had very low RNA integrity numbers (RIN, 3.7 and 4.2 respectively), and one sample (B9) had low 28S/18S peak height ratio but they were included in library preparation and sequencing. All libraries were prepared according to the dUTP strand-specific protocol, multiplexed in one 85-plex and sequenced in 5 HiSeq 2500 rapid runs to obtain paired-end, 51 bp reads.

#### Alignment of sequencing reads and quantification of gene expression

We used SNPs and indels identified in D2 (Sanger Mouse Project[50], files mgp.v3.snps.rsIDdbSNPv137.vcf and mgp.v3.indels.rsIDdbSNPv137.vcf, both on GRCm38) to construct a D2 genome and the corresponding gene annotation file using the software SeqNature[51] (version 1.2). Reads were aligned using Tophat[52] (Tophat2 version 2.0.11, Bowtie version 2.2.2.0, and Samtools version 0.1.19.0), considering either the B6 genome (GRCm38) or the D2 genome. High quality alignments were selected using the following criteria: both reads of the pair had to uniquely map to the same chromosome and within 2.3 Mb of each other (length of the longest gene). Expression levels were quantified at the gene level using HTSeq[53] (version 0.6.1), considering high quality alignments only and UCSC annotations (GRCm38/mm10).

#### Pre-processing and quality control

Raw expression counts were adjusted for library size using the R package DESeq2[54] (version 1.2.10). Out of 23,420 genes, we considered 13,271 genes with at least 10 (library size normalized) reads in at least 40% of the samples. Gene expression levels were normalized using the Box-Cox transformation as done for the organismal phenotypes.

We identified and excluded genes whose expression was affected by the order of sacrifice: at the end of our experiment, we sacrificed each cage mate in turn with an interval of about 15 minutes between the first and the second cage mate. The cage mate that was sacrificed second was in the room and we found evidence that the expression of a small number of genes was activated during this interval (i.e. their expression was correlated with order of sacrifice within cage mate pair). Some of the affected genes were well-known immediate early genes. In our experiment, this effect was confounded with potential social genetic effects as B6 mice in BD cages were always sacrificed first and D2 mice second, while in BB cages some of the B6 mice were necessarily sacrificed first and others second, and the same applies for D2 mice in DD cages. To avoid possible false positive SGE, we took a conservative approach and excluded all genes for which the order of sacrifice within pair, in B6 mice from BB cages and D2 mice from DD cages, was associated with gene expression levels (P < 0.05 P, > 1% variance explained; 375 genes in total).

We used a principal component analysis (PCA) to identify outlier samples. The first and second principal components explained 22% and 9% of variation in gene expression, and were used to define outliers (S9 Fig). A total of 7 samples were excluded (B39, D13, D30, D9, B30, D16, D39), two of which also had very low RIN. The final gene expression dataset consisted of 11 pairs of B6 cage mates, 3 B6 mice whose B6 cage mates were not included in the final sample, 15 pairs of B6/D2 cage mates, 1 B6 mouse whose D2 cage mate was not included in the final sample, 9 pairs of D2 cage mates, and 4 D2 mice whose cage mates were not included in the final sample. This amounts to 25, 16, 15, and 22 mice in the BB, BD, DB, and DD groups respectively.

In addition to removing outlier samples, we used two approaches to adjust for observed and hidden covariates affecting the expression profiles. First, for significance tests of SGE on gene expression levels, we used PEER to account for hidden sources of variation[55]; we ran PEER while including strain, strain of the cage mate, and interaction between them as observed covariates so as not to remove the genetic signal (see below), and used the expression residuals calculated from the first 5 PEER factors for statistical tests. The p-values obtained with PEER residuals were calibrated whereas other normalization strategies led to severely deflated test statistics, supporting that the PEER factors accounted for unwanted variation such as hidden batch effects.

Second, for variance partitioning and model selection, we regressed out observed technical covariates only as removal of hidden factors can reduce the residual noise. We considered several technical covariates: initial RNA concentration (as an indicator of the size of tissue dissected, since the same volume of buffer was added to all), RIN, ratio between heights of 28S and 18S peaks, proportion of first reads mapping—as they should—to the strand opposite to the feature (i.e. strand specificity of the protocol, calculated by running htseq-count a second time with the–-stranded = yes option), insert size, yield, proportion of reads mapped to the (B6 or D2) genome, proportion of mapped reads that are of high quality (as defined above), proportion of high quality reads that are exonic, number of alignments with 0, 1, or 2 errors (as calculated by SAMStat[56] version 1.09), and the following measures calculated from the output of FastQC[57] (version 0.10.1): average per base sequence quality, average per base sequence (G nucleotide used) content, average per base GC content, average per sequence GC content, average per base N content, average per sequence quality scores, average sequence duplication levels, presence or absence of overrepresented sequences. We calculated the correlation of these covariates with the first principal components, and found that *proportion of selected read pairs that are exonic* was significantly correlated with the first principal component (Pearson correlation, R^2^ = 0.66, P = 8.10^−12^). Therefore we regressed the effect of this covariate out of the expression of each gene, and used the residuals for variance partitioning and model selection.

#### Statistical modelling

Conceptually we distinguish between the ***focal individual*** (f), whose phenotype is the ***phenotype of interest*** and the dependent variable in the model, and the focal individual’s ***social partners***–here its cage mate (cm)–which may influence the phenotype of the focal individual through a number of unknown ***mediating phenotypes***. No prior knowledge or measurement of these mediating phenotypes is required as they are captured through their genetic and environmental components (social genetic effects, SGE, and social environmental effects, SEE, respectively).

Genetic effects considered in this experiment include:

- direct genetic effects (DGE, effects of the strain of the focal animal on its own phenotype–the phenotype of interest). This component is only relevant when both B6 and D2 focal mice are analysed together.
- social genetic effects (SGE, effects of the strain of the cage mate on the focal animal’s phenotype; SGE act through mediating phenotypes of the cage mate).
- an interaction between DGE and SGE (whereby the effect of the strain of the cage mate depends on the strain of the focal individual). This component is only relevant when both B6 and D2 focal mice are analysed together.

Genetic effects were modelled as fixed effects.

“Environmental” (i.e. unexplained or residual) variance may arise from:

- direct environmental effects (DEE, effects of the “environment” of the focal animal on its own phenotype)
- social environmental effects (SEE, effects of the environment of the cage mate on the focal animal’s phenotype; SEE are the environmental component of the mediating phenotypes, so whenever social effects are considered both SGE and SEE need to be accounted for)
- cage effects (which affect the phenotype of interest of all mice sharing a cage in the same way)

Environmental effects were modelled as random effects.

The model of phenotypic variation that includes all of the genetic and environmental effects listed above (note: a simpler model was effectively used for analysis, as explained next) is the following:

$$y_{f}=A_{D,f}+A_{S,cm}+A_{D,f}:\;A_{S,cm}+e_{D,f}+\underline{Z_{f}}\underline{e_{S}}+\underline{W_{f}}\underline{c}$$

where *y*_*f*_ is the phenotype of interest of the focal animal *f*; *A*_*D*,*f*_ and *A*_*S*,*cm*_ respectively refer to additive DGE of the focal individual and additive SGE from the cage mate *cm*; *A*_*D*,*f*_ : *A*_*S*,*cm*_ refers to the interaction between *A*_*D*,*f*_ and *A*_*S*,*cm*_; $\underline{Z_{f}}$ is a row of the matrix *Z* that indicates cage mates (importantly *Z*_*i*,*i*_ = 0); $\underline{e_{D}}$ is the vector of random DEE and $\underline{e_{S}}$ the vector of random SEE. $\underline{W_{f}}$ is a row of the matrix *W* that indicates cage assignment and *c* the vector of cage effects.

The joint distribution of the environmental random effects is defined as:

$$\left[\begin{array}{c} \underline{e_{D}}\\ \underline{e_{S}}\\ \underline{c} \end{array}\right]∼MVN(0,\left[\begin{array}{ccc} \sigma_{E_{D}}^{2}I & \sigma_{E_{DS}}I & 0\\ \sigma_{E_{DS}}I^{T} & \sigma_{E_{S}}^{2}I & 0\\ 0 & 0 & \sigma_{C}^{2}I \end{array}\right])$$

The covariance $\sigma_{E_{DS}}$ between the random direct and social environmental effects exists because an environmental factor may influence both the phenotype of interest and the mediating traits, in which case it will influence the phenotype of interest both in a direct ($\underline{e_{D}}$) and an indirect ($\underline{e_{S}}$) manner. Depending on 1) the direction of the environmental effect on the phenotype of interest, 2) the directions of the effects on the mediating traits, and 3) the signs of the correlations between mediating traits and phenotype of interest, $\sigma_{E_{DS}}$ may be positive or negative.

The environmental covariance is:

$$\begin{array}{l} cov\;(e_{i},e_{j})=\sigma_{E_{D}}^{2}+\sigma_{E_{S}}^{2}+\sigma_{C}^{2}\;\mathrm{if}\;i=j\\ cov\;(e_{i},e_{j})=2\;\sigma_{E_{DS}}+\sigma_{C}^{2}\;\mathrm{if}i\neq j\;\mathrm{and}\;i\;\mathrm{and}\;j\;\mathrm{share}\;a\;\mathrm{cage}\\ cov\;(e_{i},e_{j})=0\;\mathrm{if}\;i\;\mathrm{and}\;j\;\mathrm{are}\;\mathrm{in}\;\mathrm{different}\;\mathrm{cages} \end{array}$$

Note that *cov* (*e*_*i*_,*e*_*j*_) may be negative as a result of $\sigma_{E_{DS}}$ being negative.

When group size is constant, as is the case in this dataset (group size = 2), *e*_*D*_, *e*_*S*_, and *c* are not identifiable. Thus we instead used a single random environmental term, whose correlation structure is described below, as has been done in other studies of social genetic effects[25]:

$$\begin{array}{l} cov\;(e_{i},e_{j})=\;\sigma_{E}^{2}\;\mathrm{if}\;i=j\\ cov\;(e_{i},e_{j})=\;\rho \sigma_{E}^{2}\;\mathrm{if}\;i\neq j\;\mathrm{and}\;i\;\mathrm{and}\;j\;\mathrm{share}\;a\;\mathrm{cage}\\ cov\;(e_{i},e_{j})=\;0\;\mathrm{if}\;i\;\mathrm{and}\;j\;\mathrm{are}\;\mathrm{in}\;\mathrm{different}\;\mathrm{cages} \end{array}$$

From the expressions of *cov* (*e*_*i*_,*e*_*j*_) above we see that $\sigma_{E}^{2}\;=\;\sigma_{E_{D}}^{2}+\;\sigma_{E_{S}}^{2}+\;\sigma_{C}^{2}$ and $\rho \;=\;(2\;\sigma_{E_{DS}}\;+\;\sigma_{C}^{2})/\sigma_{E}^{2}\;$. As the determinant of the environmental covariance matrix is necessarily positive, −1 ≤ *ρ* ≤ 1.

Note that under the assumption 0 ≤ *ρ* ≤ 1 the environmental component could be rewritten as a sum of cage effects and iid residuals. This assumption is often made when group size is large (e.g. [2]) because

$$\rho =\left(2\;\sigma_{E_{DS}}+(n-2)\sigma_{E_{S}}^{2}+\sigma_{C}^{2}\right)/\sigma_{E}^{2}\;$$

where *n* is the group size. $(n-2)\sigma_{E_{S}}^{2}$ is likely to be positive and large for large groups, so *ρ* is likely be positive for large groups. However, in this experiment group size is 2 so $(n-2)\sigma_{E_{S}}^{2}\;=\;0$ and *ρ* may well be negative. Thus we cannot rewrite the residual term as a sum of cage effects and iid residuals.

To investigate interactions between DGE and SGE (see Methods sections *Model selection* and *Variance partitioning* below), we considered all mice (B6 and D2) as focal individuals. For all other analyses we analysed B6 focal mice and D2 focal mice separately. We did so because there was evidence that interactions exist between DGE and SGE (results reported in the columns under “All mice” in S2 Table and shown in S2 Fig) and analysing B6 and D2 focal mice separately in that case simplified results interpretation.

When analysing B6 and D2 focal mice separately, we used the following simpler model:

$$\begin{array}{l} y_{f}\;=\;A_{S,cm}\;+\;e_{f}\\ \;=\;\beta_{S}X_{cm}+e_{f} \end{array}$$ (1)

where *β*_*S*_ is the regression coefficient of SGE and *X*_*cm*_ the strain of the cage mate *cm*, with strain encoded as 0 for B6 and 1 for D2.

#### Model selection

To investigate whether interactions between DGE and SGE might contribute to phenotypic variation, we sought to identify the combination of genetic and environmental terms from {DGE, SGE, interaction between DGE and SGE, DEE, SEE, covariance between DEE and SEE, and cage effects} that best modelled phenotypic variation. All mice (B6 and D2) were included as focal individuals for this analysis. Constraints were imposed on the environmental correlation parameter *ρ* when SEE and/or cage effects were not included in the model. Thus, the models were fitted in R using either the functions lm (stats package), lme (nlme package), or gls (nlme package, corCompSymm correlation structure for the residuals), as appropriate. The best model was selected using the Akaike information criterion (AIC) calculated based on the maximum-likelihood (ML) fit using the AIC function in the R stats package. The results of this analysis are reported in S2 Fig and the columns under “All mice” in S2 Table).

#### Variance partitioning

For S2 Fig and the columns under “All mice” in S2 Table, both B6 and D2 mice were considered as focal individuals and we used the following model:

$$\begin{array}{l} y_{f}=A_{D,f}+A_{S,cm}+A_{D,f}:\;A_{S,cm}+e_{f}\\ \;=\;\beta_{D}X_{f}+\beta_{S}X_{cm}+\beta_{D:S}X_{f}.X_{cm}+e_{f} \end{array}$$ (2)

where *β*_*D*_ is the regression coefficient of DGE, *X*_*f*_ the strain of *f*, and *β*_*D*:*S*_ the regression coefficient of the interaction between DGE and SGE. *X*_*f*_.*X*_*cm*_ is the product of the numerically encoded strains of *f* and *cm*, and therefore was 0 except for D2 focal mice with D2 cage mates.

All other results were obtained by analysing B6 and D2 focal mice separately using Model (1).

All models were fitted using restricted maximum likelihood (REML). The proportion of phenotypic variance explained by each fixed effect in the model (Model (1) or Model (2)) was calculated as the ratio of the sample variance of that term (i.e. the expected value of the variance of that term, which is $\mathrm{var}(\beta_{S}\underline{X})$ for SGE) and the sample phenotypic variance. We obtained the sample phenotypic variance by drawing from the fitted model and calculating the average variance of the draws.

Standard errors were obtained using Gaussian propagation of uncertainty.

#### Statistical significance tests

Statistical significance analyses for SGE were carried out in B6 and D2 focal mice separately (S1 Fig), based on Model (1). Significance of the SGE covariate was assessed using a likelihood ratio test. Q values[58] implemented in the R package qvalue [59] were used to adjust for multiple testing across traits and focal strain (43 traits x 2 focal strains, 86 tests in total).

#### Gene set enrichment analysis

We considered B6 and D2 focal mice separately for this analysis, using the R package topGO[60] and the annotation package org.Mm.eg.db to perform gene set enrichment analyses. We used the residuals from PEER and considered the variance explained by SGE in Model 1 as score for each gene. Enrichment P values were obtained with the Kolmogorov-Smirnov test and the *elim* algorithm that decorrelates the GO graph structure[61]. Q values[58] were used for multiple testing correction, and obtained using the R package package[59].

### Outbred mice dataset

#### Description of the dataset

We used the raw data from Valdar *et al*.[22] (genotypes and organismal phenotypes), Huang *et al.[23]* (organismal phenotype: cellular proliferation in the subgranular zone of the dentate gyrus, a measure of adult neurogenesis) and Huang *et al.[24]* (gene expression in the hippocampus), which were collected on Heterogeneous Stock (HS) mice. HS mice are descended from eight inbred progenitor strains through more than 50 generations of circular breeding with 24 families[62]. As a result, each HS mouse is genetically unique. The mice included in this dataset are from a colony established at Oxford for a few generations, and for which pedigree data are available (3,149 mice). Cage information was available for a subset of 2,448 mice, filling 549 cages in groups of 2 to 7 mice (S4 Fig). Genotypes at 13,459 single nucleotide polymorphisms were available for a subset of 1,940 of those 2,448 mice, and a pair-wise genetic similarity matrix shows that they were related at different levels (S8 Fig), including (but not only) many full siblings. Some phenotype data were available for all 2,448 mice, but the number of mice phenotyped for each organismal phenotype is reported in S5 Table.

#### Inference of relationship coefficients using the single-step or H matrix

Our analysis of this dataset is based on linear mixed models, which use a genetic covariance matrix made of pair-wise relationship coefficients. These coefficients can be estimated from genome-wide genotypes, however as 508 cage mates of phenotyped mice had not been genotyped, we estimated relationship coefficients for all 3,149 mice based on both genotype and pedigree data.

We discarded the genotypes of 18 mice because they represented likely duplicates.

We infered the pair-wise relationship coefficient of the 1,227 non-genotyped mice with all 3,149 mice using the single-step or H matrix[63–65], which was constructed as (in the inverse scale;):

$$H^{-1}=A^{-1}+\left(\begin{array}{cc} 0 & 0\\ 0 & G^{(*)-1\;}+A_{gg}^{-1} \end{array}\right)$$

where *A*^−1^ is the inverse of pedigree-based Wright (1921) relationship matrix, and *A*_*gg*_ is the pedigree-based relationship submatrix across genotyped individuals. The pedigree consisted of the genotyped individuals and their parents, some of them common across sibships. To warrant that both *G*^(*)^ and *A*^−1^ refer to the same base population and to ensure that *G*^(*)^ is a valid covariance matrix, *G*^(*)^ was constructed as [66, 67]

$$G^{\left(*\right)}=0.95\left(a+b\frac{M\prime M}{2\sum (p_{i}q_{i})}\right)+0.05A_{gg}$$

where *a* and *b* are constants (respectively 0.015 and 0.982) based on statistics of *A* and *G* so that average relationship and average inbreeding agree for both matrices. These constants correct for the random drift and the loss on heterozygosity across generations. Matrix *M* contains centered genotypes based on observed allelic frequencies *p* and *q* = 1 − *p* for each locus as in [68]. The matrix *H* was obtained by inversion of *H*^−1^. Program pregsf90 ([69]; available at http://nce.ads.uga.edu/wiki/doku.php) was used to construct *H*. Using this matrix, all estimates of genetic variance refer to the pedigree base populations, i.e. the parents of the animals in the study. Because this is only one generation back in time, we can assume that this base genetic variance is concordant with the genetic variance in the phenotyped population.

#### Processing of organismal phenotypes

We considered a subset of the experimental variables recorded by Valdar *et al*.[22] as covariates for our analysis (S5 Table). Each phenotype was normalized using a covariate-aware Box-Cox transformation[49], and the covariates were fitted as fixed effects in the models used in our analyses.

#### Re-processing of expression data

Gene expression in the hippocampus was assessed by hybridization of mRNA to the Illumina Mouse WG-6 v1 BeadArray platform. The data, as originally pre-processed by Huang et al. had critical shortcomings: S10A and S10B Fig show that the higher correlation that can be seen in the raw data between probes mapping to the same gene compared to random probe pairs is lost in the pre-processed data from Huang *et al*.[24]. We re-processed the data as detailed in Section 2.6 of Krohn[70]. Briefly, the BeadArray images were imported into the Gene Expression module (V 1.6.0) of the Illumina GenomeStudio (V 2010.1) without invoking any data adjustment procedures. These raw data were transferred to R using the Bioconductor package lumi[71]. A cluster dendrogram enabled identification of four outlier samples, which were excluded from further pre-processing. Two thousand control probes scattered across the Illumina chip enabled subtraction of background noise levels via lumi. Standardisation of variance and mean were carried out via lumi’s variance stabilising transformation and robust spline normalisation. Then a series of four filters were applied to eliminate unreliable probes: following the pipeline developed by Barbosa-Morais *et al*.[72], probes not rated as “perfect” (50 out of 50 nucleotides match the reference genome) or “good” (48 to 49 out of 50 matches to the genome) were removed; so were probes that mapped to more than one genomic location according to BLAST queries[73] and probes that included a SNP (NCBI dbSNP Build 137); finally, only probes detected in at least 5% of the mice at a 0.95 detection level (as per GenomeStudio) were retained. Following filter application, 15,736 of the original 47K probes, mapping to 11,996 genes, were kept. Finally, ComBat[74] was used to control for batch effects (the data were assayed over several months). S10C Fig shows that our processing of the data kept the biological correlation that exists between probes mapping to the same gene.

#### Statistical models

We considered a linear additive model,

$$y_{f}=\underline{X_{f}}\underline{b}+a_{D,f}+e_{D,f}+\underline{Z_{f}}\underline{a_{S}}+\underline{Z_{f}}\underline{e_{S}}+\underline{W_{f}}\underline{c}$$ (3)

Here, *y*_*f*_ is the phenotypic value of the focal mouse *f*, $\underline{X_{f}}$ is a row of the matrix *X* of covariate values and *b* a column vector of corresponding estimated coefficients. *a*_*D*,*f*_ is the additive direct genetic effects (DGE), also called breeding value, of *f*. $\underline{Z_{f}}$ is a row of the matrix *Z* that indicates cage mates (importantly *Z*_*i*,*i*_ = 0) and $\underline{a_{S}}$ the column vector of additive social genetic effects (SGE). $\underline{e_{D}}$ refers to direct environmental effects (DEE) and $\underline{e_{S}}$ to social environmental effects (SEE). $\underline{W_{f}}$ is a row of the matrix *W* that indicates cage assignment and *c* the column vector of cage effects

Because the genetic setup is not binary (B6 / D2 in the experiment with inbred strains) but individuals are genetically unique and related to varying degrees, we modeled the components in Model (3) as random effects, while accounting for covariance between different effects. The joint distribution of all random effects is defined as:

$$\left[\begin{array}{c} \underline{a_{D}}\\ \underline{a_{S}}\\ \underline{e_{D}}\\ \underline{e_{S}}\\ \underline{c} \end{array}\right]∼\text{MVN}\;(\;0,\;\left[\begin{array}{ccccc} \sigma_{A_{D}}^{2}H_{1} & \sigma_{A_{DS}}H_{2} & 0 & 0 & 0\\ \sigma_{A_{DS}}{\text{H}}_{2}^{T} & \sigma_{A_{S}}^{2}H_{3} & 0 & 0 & 0\\ 0 & 0 & \sigma_{E_{D}}^{2}I_{1} & \sigma_{E_{DS}}I_{2} & 0\\ 0 & 0 & \sigma_{E_{DS}}{\text{I}}_{2}^{T} & \sigma_{E_{S}}^{2}I_{3} & 0\\ 0 & 0 & 0 & 0 & \sigma_{C}^{2}I_{4} \end{array}\right]$$

and the phenotypic covariance is:

$$cov\;(y_{i},y_{j})=\sigma_{A_{D}}^{2}\;{H_{1}}_{i,j}+\sigma_{A_{DS}}\;\{{\left(H_{2}Z^{T}\right)}_{i,j}+{\left(ZH_{2}^{T}\right)}_{i,j}\}+{\sigma_{A_{S}}^{2}(ZH_{3}Z^{T})}_{i,j}+\sigma_{E_{D}}^{2}\;{I_{1}}_{i,j}+\sigma_{E_{DS}}\;\{{\left(I_{2}Z^{T}\right)}_{i,j}+{\left(ZI_{2}^{T}\right)}_{i,j}\}+{\sigma_{E_{S}}^{2}(ZI_{3}Z^{T})}_{i,j}+\sigma_{C}^{2}\;{\left(WI_{4}W^{T}\right)}_{i,j}$$ (4)

*H*_1_, *H*_2_, and *H*_3_ were calculated by dividing the (single-step) *H* matrix by, respectively, *sampleVar*(*H*), $\sqrt{sampleVar(H)\;\times \;sampleVar(ZHZ^{T})\;}$, and *sampleVar*(Z*HZ*^*T*^), where *sampleVar* is the sample variance of the corresponding covariance matrix: suppose that we have a vector $\underline{x}$ of random variables with covariance matrix *M*, the sample variance of *M* is calculated as

$$sampleVar(M)=\frac{Tr(PMP)}{n-1}$$

*Tr* denotes the trace, *n* is the sample size, and $P\;=\;I-\frac{11^{\prime }}{n}$ [75, 76].

As a result of this scaling, $\sigma_{A_{D}}^{2}$, $\sigma_{A_{DS}}$, and $\sigma_{A_{S}}^{2}$ directly reflect the relative contribution of DGE, the covariance between DGE and SGE, and SGE.

Similarly, *I*_1_, *I*_2_, and *I*_3_ were calculated by dividing the identity matrix *I* by, respectively, *sampleVar*(*I*) = 1, $\sqrt{sampleVar(I)\;\times \;sampleVar(ZIZ^{T})\;}$, and *sampleVar*(*ZIZ*^*T*^), so that $\sigma_{E_{D}}^{2}$, $\sigma_{E_{DS}}$, and $\sigma_{E_{S}}^{2}$ directly reflect the phenotypic variance explained by DEE, the covariance between DEE and SEE, and SEE.

*I*_4_ was calculated by dividing the identity matrix I by *sampleVar*(*WIW*^*T*^).

See S1 Note for a discussion of how these models differ from published SGE models.

#### Variance partitioning

For each trait, Eq (4) was used to partition phenotypic variance into its individual components. All models were fitted using LIMIX[26, 27], which we extended to handle models with correlated variance components. The model was fitted using restricted maximum likelihood (REML). We report the contribution of DGE and SGE to phenotypic variation as the proportions of phenotypic variance explained by DGE and SGE, i.e. the ratio between the sample variance explained by DGE ($\sigma_{A_{D}}^{2}$) or SGE ($\sigma_{A_{S}}^{2}$) and the sample variance of the overall phenotype covariance matrix (defined by Eq 4, with all variance components estimated).

Standard errors on variance components are estimated as the square root of the corresponding diagonal entries of the inverse of the Fisher information, which is the negative expected value of the Hessian[77].

#### Simulation experiments

Phenotypes were simulated based on the genotypes and the cage relationships of the full set of 2,448 mice. Phenotypes were drawn from model (3). We note $\rho_{A_{DS}}$ the correlation between $\underline{a_{D}}$ and $\underline{a_{S}}$: $\rho_{A_{DS}}\;=\;\sigma_{A_{DS}}\;/\left(\sigma_{A_{D}}\;\times \;\sigma_{A_{S}}\right)$.

We present several sets of simulations:

- one based on the estimated contributions of all parameters averaged over all organismal phenotypes: $\sigma_{A_{D}}^{2}$ = 15%, $\sigma_{A_{S}}^{2}$ = 4%, $\rho_{A_{DS}}$ = 0.5, $\sigma_{E_{D}}^{2}$ = 52%, $\sigma_{E_{D}}^{2}$ = 0, $\rho_{E_{DS}}$ = 0, $\sigma_{C}^{2}$ = 22% (S6B Fig and S7B Fig)
- one based on the estimated contribution of all parameters averaged over the six phenotypes with highest SGE: $\sigma_{A_{D}}^{2}$ = 5%, $\sigma_{A_{S}}^{2}$ = 27%, $\rho_{A_{DS}}$ = 0.92, $\sigma_{E_{D}}^{2}$ = 5%, $\sigma_{E_{D}}^{2}$ = 0, $\rho_{E_{DS}}$ = 0, $\sigma_{C}^{2}$ = 51% (S6C Fig and S7C Fig)
- $\sigma_{A_{D}}^{2}$ taking values {0,10,20,30,40,50} with all other parameters set to their average estimated contribution across all phenotypes (so $\sigma_{A_{S}}^{2}$ = 4%, $\rho_{A_{DS}}$ = 0.5, $\sigma_{E_{D}}^{2}$ = 52%, $\sigma_{E_{S}}^{2}$ = 0, $\rho_{E_{DS}}$ = 0, $\sigma_{C}^{2}$ = 22%) (S6A Fig and S7A Fig)
- $\sigma_{A_{S}}^{2}$ taking values {0,10,20,30,40,50} with all other parameters set to their average estimated contribution across all phenotypes (Fig 2C, S6A Fig and S7A Fig)
- $\rho_{A_{DS}}$ taking values {-0.8,-0.4,-0.2,0.2,0.4,0.8} with all other parameters set to their average estimated contribution across all phenotypes (S6A Fig and S7A Fig)
- $\sigma_{E_{D}}^{2}$ taking values {0,10,20,30,40,50} with all other parameters set to their average estimated contribution across all phenotypes (S6A Fig and S7A Fig)
- $\sigma_{E_{S}}^{2}$ taking values {0,10,20,30,40,50} with all other parameters set to their average estimated contribution across all phenotypes (S6A Fig and S7A Fig)
- $\rho_{E_{DS}}$ taking values {-0.8,-0.4,-0.2,0.2,0.4,0.8} with all other parameters set to their average estimated contribution across all phenotypes (S6A Fig and S7A Fig)
- $\sigma_{C}^{2}$ taking values {0,10,20,30,40,50} with all other parameters set to their average estimated contribution across all phenotypes (S6A Fig and S7A Fig)

500 simulations were drawn for each combination of parameters.

#### Statistical significance tests

Statistical significance was assessed using restricted likelihood ratio tests, comparing the full model in Eq (3) to a null model without the SGE term. Because both SGE and the covariance between DGE and SGE are constrained to zero in the null model, we calculated significance by comparing the log likelihood ratio statistics to the χ^2^ distribution with 2 degrees of freedom, which is conservative. Q values[58] were used for multiple testing correction, and obtained using the R package qvalue [59].

## Accession numbers

Gene expression data from the experiment with inbred strains are available from ArrayExpress E-MTAB-5276. Phenotype data for the same experiment are provided as S7 Table.

## Supporting Information

S1 Note Differences between our models and those published in the field of animal breeding. (DOCX)

S1 Fig Experimental design and analysis approach (experiment with inbred strains). (A) When jointly analyzing B6 and D2 focal mice (for S2 Fig and columns “all mice” of S2 Table), DGE, SGE and their interaction can be detected. All other results were obtained by considering B6 focal mice (B) and D2 focal mice (C) separately. The only genetic parameter then is the strain of the cage mate. Environmental effects are not shown.
(TIF)

S2 Fig Evidence for interactions between DGE and SGE in the experiment with inbred strains. The contribution to phenotypic variance of DGE, SGE, and their interaction is shown. All organismal phenotypes are shown (see S1 Table for a description of each measure). They are plotted in order of decreasing contribution of the interaction component. Blue stars indicate phenotypes for which a model with interaction was selected based on the Akaike information criterion (AIC).
(TIF)

S3 Fig Consistent direction of social genetic effects across the anxiety phenotypes that are significantly affected by SGE in D2 focal mice (experiment with inbred strains). Box plots for the six anxiety-related phenotypes that are significantly affected by SGE in D2 mice (P < 0.05, see Table 1). The raw data are shown, for all four types of cages (B6 focal mice with B6 cage mate (BB), B6 focal mice with D2 cage mate (BD), D2 focal mice with B6 cage mate (DB), and D2 focal mice with D2 cage mate (DD).
* The sign of the correlation between anxiety and time spent not moving in the center of the activity chamber in the first minute of the test in unclear: greater time spent in the center of the apparatus usually indicates lower anxiety levels, but the measure may reflect the mouse freezing where it was dropped off in the apparatus–which would be a sign of high anxiety.
(EPS)

S4 Fig Group sizes in the outbred mice dataset. (EPS)

S5 Fig Consistent results using genomic and inferred relationship coefficients (outbred mice dataset). Comparison of the contribution of DGE (A) and SGE (B) to organismal phenotypes, estimated either using genotype and pedigree data (see Methods; all mice included, x axis) or genotype data only (subset of mice that were in fully genotyped cages, y axis).
(EPS)

S6 Fig Need to account for SGE to obtain unbiased DGE estimates in the outbred mice dataset. DGE estimates were evaluated using simulated phenotypes: the difference between estimated and simulated DGE is shown on the y-axis. Each boxplot summarizes the results from 500 simulations, and the whiskers extend to the 1^st^ and 9^th^ deciles of the distribution. (A) Phenotypes simulated with varying levels DGE, SGE, correlation between DGE and SGE, DEE, SEE, correlation between DEE and SEE, and cage effects (top to bottom panels). (B) Phenotypes simulated based on the estimated contributions of all parameters averaged over all organismal phenotypes. (C) Phenotypes simulated based on the estimated contributions of all parameters averaged over the six organismal phenotypes with strongest SGE. The boxplots in blue correspond to results from the full model with DGE, SGE SEE and cage effects, those in green correspond to the model with DGE and cage effects only, and those in red correspond to the model with DGE only.
(EPS)

S7 Fig Unbiased SGE estimates in the outbred mice dataset. SGE estimates were evaluated using simulated phenotypes: the difference between estimated and simulated SGE is shown on the y-axis. Each boxplot summarizes the results from 500 simulations, and the whiskers extend to the 1^st^ and 9^th^ deciles of the distribution. (A) Phenotypes simulated with varying levels DGE, SGE, correlation between DGE and SGE, DEE, SEE, correlation between DEE and SEE, and cage effects (top to bottom panels). (B) Phenotypes simulated based on the estimated contributions of all parameters averaged over all organismal phenotypes. (C) Phenotypes simulated based on the estimated contributions of all parameters averaged over the six organismal phenotypes with strongest SGE. Estimates were obtained from the full model with DGE, SGE, and cage effects.
(EPS)

S8 Fig Above-average relatedness of cage mates compared to non-cage mates in the outbred dataset. (EPS)

S9 Fig Identification of outlier gene expression profiles using principal component analysis in the experiment with inbred strains. (A) Variance explained by the principal components. (B) Scatterplot of the first and second principal components. Samples that were excluded from all analyses (outliers) are highlighted.
(EPS)

S10 Fig Improved quantification of gene expression in the hippocampus following re-processing of the data (outbred mice dataset). The quality of the quantification is evaluated based on the correlation between probes that target the same gene (orange line) and probes that target different genes (blue line). The empirical cumulative function of these correlations is shown, so a smaller area under the curve is indicative of greater correlations.
(A) Raw data from Huang *et al*.^26^: expression values from pairs of probes that map to the same gene are more highly correlated than expression values from pairs of probes that map to different genes.
(B) Pre-processed data from Huang *et al*.^26^: same-gene probes are only as correlated as pairs of probes mapping to different genes, suggesting a loss of biological signal.
(C) Data pre-processed by us: same-gene probes are more highly correlated than different-gene probes, suggesting biological signal is retained.
(EPS)

S11 Fig Independency of residual variability on group size (outbred mice dataset). The residuals from a model with covariates fitted as fixed effects only (y axis) were plotted as a function of group size (x axis). Only the six organismal phenotypes with strongest SGE are shown.
(EPS)

S12 Fig Consistent results using mice in cages of 4,5 or 6 mice only (outbred mice dataset). Comparison of the contribution of DGE (A) and SGE (B) to organimal phenotypes, estimated either using all mice (x axis) or only those mice that were in groups of size 4, 5 or 6 (y axis).
(EPS)

S1 Table Organismal phenotypes collected in the experiment with inbred strains. (XLS)

S2 Table SGE significance and effect sizes for all organismal phenotypes measured in the experiment with inbred strains. (XLSX)

S3 Table SGE significance for non-residualized organismal phenotypes measured in the experiment with inbred strains. (XLSX)

S4 Table SGE significance and effect sizes for all genes expressed in the prefrontal cortex (experiment with inbred strains). (XLS)

S5 Table Description of all organismal phenotypes in the outbred mice dataset, and significance and effect sizes of SGE. (XLSX)

S6 Table SGE significance and effect sizes for all genes expressed in the hippocampus (outbred mice dataset). (XLSX)

S7 Table Raw phenotype data collected on inbred strains. (XLS)
