# Supplementary material for: Genetic Variation in the Social Environment Contributes to Health and Disease
Source: PLoS Genet. 2017 Jan 25;13(1):e1006498. doi: 10.1371/journal.pgen.1006498 (PMC5266220; doi:10.1371/journal.pgen.1006498)
Supplement: S1 Note — (DOCX) [file pgen.1006498.s001.docx]

**Genetic variation in the social environment**

**contributes to health and disease**

**Supplementary note**

Amelie Baud^1*^, Megan K. Mulligan^2¶^, Francesco Paolo Casale^1¶^, Jesse F. Ingels^2^, Casey J. Bohl^2^, Jacques Callebert^3^, Jean-Marie Launay^3^, Jon Krohn^4^, Andres Legarra, Robert W. Williams^2^, Oliver Stegle^1*^

^1^ European Molecular Biology Laboratory, European Bioinformatics Institute, Wellcome Trust Genome Campus, Hinxton, Cambridge, United Kingdom

^2^ Department of Genetics, Genomics and Informatics, University of Tennessee Health Science Center, Memphis, Tennessee, USA 38163

^3^ AP-HP, Hôpital Lariboisière, Department of Biochemistry, INSERM U942, Paris F-75475, France

^4^ Wellcome Trust Centre for Human Genetics, Oxford, United Kingdom

^5^ INRA, UMR 1388 GenPhySE, 31326 Castanet Tolosan, France

* Corresponding authors

Emails: abaud@ebi.ac.uk, oliver.stegle@ebi.ac.uk

^¶^ These authors contributed equally to this work

This note aims at highlighting important differences between our models and those published in the field of animal breeding(1-6). It is not an independent document and needs to be read together with our Methods. All notation are consistent with those used in our Methods unless indicated otherwise.

- **quantities reported to quantify SGE**

In the literature SGE have been quantified either by reporting the variance components relative to SGE estimated in the “animal model” (i.e. the variance of the elements of $\underline{a_{S}}$) or by reporting the heritable variance expressed on the scale of phenotypic variance among individuals(1):

$$T^{2}= \sigma_{A_{D}}^{2}+2\left( n-1 \right)\sigma_{A_{DS}}+ {(n-1)}^{2}\sigma_{A_{S}}^{2}$$

where $n$ is the group size (assuming all groups have equal size) and $\sigma_{A_{D}}^{2}$, $\sigma_{A_{DS}}$, and $\sigma_{A_{S}}^{2}$ are the variance components estimated in the animal model. $T^{2}$ is of interest to discuss the impact of SGE on (artificial and natural) selection, but it is not the quantity of interest to us. We want to estimate the proportion of sample phenotypic variance that is explained by SGE, defined as:

$${sampleVar(M_{SGE})}/{sampleVar(M)}= {\frac{Tr(PM_{SGE}P)}{n-1}}/{\frac{Tr(PMP)}{n-1}}$$

Here, $n$ is the sample size and not the group size as above, $sampleVar$ is the sample variance(7, 8), and $M_{SGE}$ is the component of the phenotypic covariance matrix $M$ of SGE origin, highlighted in yellow in the following equation:

$$M_{i,j} = \sigma_{A_{D}}^{2} {H_{1}}_{i,j}+ \sigma_{A_{DS}} \{ {(H_{2}Z^{T})}_{i,j} + {(ZH_{2}^{T})}_{i,j} \} + {\sigma_{A_{S}}^{2}(ZH_{3}Z^{T})}_{i,j} + \sigma_{E_{D}}^{2} {I_{1}}_{i,j}+ \sigma_{E_{DS}} \{ {(I_{2}Z^{T})}_{i,j} + {(ZI_{2}^{T})}_{i,j} \} + {\sigma_{E_{S}}^{2}(ZI_{3}Z^{T})}_{i,j} + \sigma_{C}^{2} {(WI_{4}W^{T})}_{i,j}$$

We scale $H_{1}$, $ZH_{3}Z^{T}$, $I_{1}$, $ZI_{3}Z^{T}$ and $WI_{4}W^{T}$ to sample variance 1 and scale $\{ {(H_{2}Z^{T})}_{i,j} + {(ZH_{2}^{T})}_{i,j} \}$ and $\{ {(I_{2}Z^{T})}_{i,j} + {(ZI_{2}^{T})}_{i,j} \}$accordingly (see Methods) so that the estimated variance components immediately give the relative contribution of the different sources of phenotypic variation (DGE, covariance between DGE and SGE, SGE, etc.). By dividing the variance components by $sampleVar(M)$, we obtain the proportion of sample phenotypic variance explained by the different sources.

Our approach may seem unnecessary to investigators used to quantify DGE (heritability) as when DGE only are modelled (with iid residuals) and an appropriate genetic covariance matrix is used (mean of zero and a mean diagonal value of one)(9, 10), there is no need to scale the genetic covariance matrix. However, when SGE are modelled in addition to DGE and because their contribution to phenotypic covariance is of the form (see Methods):

$$\sigma_{A_{DS}} \left\{ \left( H_{2}Z^{T} \right)_{i,j} + \left( ZH_{2}^{T} \right)_{i,j} \right\}+ {\sigma_{A_{S}}^{2}\left( ZH_{3}Z^{T} \right)}_{i,j}$$

$$+ \sigma_{E_{DS}} \{ {(I_{2}Z^{T})}_{i,j} + {(ZI_{2}^{T})}_{i,j} \} + {\sigma_{E_{S}}^{2}(ZI_{3}Z^{T})}_{i,j}$$

some scaling is required for the estimated variance components to reflect the relative contribution of the different sources of phenotypic variation.

- **variability depending on group size**

A subset of published studies have considered residuals with group-size dependent variance (heteroskedastic residuals). In particular, the variance of $e_{D}$ has been allowed to differ depending on group size(6).

We did not do so in our study for the following two reasons. First, the residuals of a model with fixed effect covariates only did not show any dependency on group size (S11 Fig), and results from restricting the dataset to only those cages with 4, 5 or 6 mice (2,148 out of 2,448 mice) were consistent with results from the full dataset (S12 Fig), suggesting that heterogeneity in variance between group sizes is not an issue in this study. Secondly, allowing for different variances of Ed would add as many parameters as there are different group sizes (6 in our dataset) to the model. Actually, if we did allow for different variances for $e_{D}$, we would also need to allow for different variances for $e_{S}$ (seen in off diagonal elements in the residual covariance matrix), which means the model would have an additional [2 x number of different group sizes] parameters (12 in our dataset). While this approach would be more general the excess number of parameters is not desirable and this approach would certainly not scale easily to other datasets with more different group sizes.

- **dilution of social effects (SGE and SEE)**

It has been proposed that dilution of social effects should be modelled to reflect the fact that an individual’s social effect (SGE + SEE) on a single recipient decrease when group size increases (2, 6). However, we did model dilution for a number of reasons. Firstly, results from restricting the dataset to only those cages with 4, 5 or 6 mice (2,148 out of 2,448 mice) were consistent with results from the full dataset (S2 Fig), suggesting there is no need to account for dilution in our study. Secondly, there isn’t any limiting condition (such as finite amount of food) that we can think of that would affect the phenotypes included in this dataset (in particular those most affected by SGE). Second, it is possible that mechanisms exist whereby individual social effects first increase with group size before decreasing (e.g. dominance in groups of 2 mice may be easily established so that there is little fighting and little associated social effects, whereas social hierarchies in larger groups may be more difficult to establish which would result in more fighting and more associated social effects; in much larger groups there could be multiple dominant mice thereby breaking the linear dependency between social effects and group size); such non linear dependency on group size would not be captured by a “dilution” function, yet using a more general function is not really desirable (see reason 3). Third, we are concerned that the introduction of the additional dilution parameter renders the estimation of p-values more challenging. For example, (2) and (6) modelled dilution as

$Z_{S\left( d \right)}\left( i,j \right)=\left( \frac{\bar{n}-1}{n-1} \right)^{d}$ when $i$ and $j$ are cage mates

$Z_{S\left( d \right)}\left( i,j \right)=0$ otherwise

$\bar{n}$ denotes average group size. The additional parameter $d$ was optimized on the alternative model before the alternative and null model (without SGE) were compared to calculate the significance of SGE. The p-values thus obtained would be anti-conservative.

- **explicit modelling of non-genetic effects (**$\boldsymbol{e}_{\boldsymbol{D}}$**,** $\boldsymbol{e}_{\boldsymbol{S}}$**, and** $\boldsymbol{c}$**)**

Published models usually do not model non-genetic effects explicitly: rather than modelling random DEE ($e_{D}$), SEE ($e_{S}$), and cage effects ($c$), they either use a random group effect plus iid residuals(1) or use a single non-genetic term $e^{*}$ with correlation structure defined by $corr\left( e_{i}^{*},e_{i}^{*} \right)=1$, $corr\left( e_{i}^{*},e_{j}^{*} \right)=r_{\left( n \right)}$ if $i$ and $j$ are group mates (the group size is $n$, $r$ is a function of $n$, and $-1 \leq r \leq1$), and $corr\left( e_{i}^{*},e_{j}^{*} \right)=0$ otherwise(4). A model with random group effect plus iid residuals is used when the non-genetic covariance between any two individuals is assumed to be always positive (i.e. either $\sigma_{E_{DS}} \geq0$ or all group sizes are large enough(1)). A model with $e^{*}$ on the contrary allows some non-genetic covariances to be negative (when $r <0$).

Because mice are typically housed in small groups (2 to 7 mice per cage in the outbred mice dataset), we wanted to allow the non-genetic covariance to be negative, and would therefore not have used a model with random group effect plus iid residuals.

We could have used a model with $e^{*}$ but did not do so for two reasons: first, a model with explicit $e_{D}$, $e_{S}$, and $c$ is more constrained than a model with $e^{*}$. In particular the relationship between residual variance and group size is constrained to be linear, and should dilution be modelled, the dilution function and parameter would be constrained to be the same for $a_{S}$and $e_{S}$. A model with $e^{*}$ on the contrary has a very general form with two parameters (non-genetic variances and covariances) per group size. Fitting such a general model would not scale well (with the number of different group sizes), thus requiring vey large datasets.

The second reason for explicitly modelling $e_{D}$, $e_{S}$, and $c$ is that it leaves open the possibility to investigate these parameters and learn about social effects of non-genetic origin.

However, when non-genetic effects are modelled explicitly, they can be non identifiable in some cases, in particular but not only when group size is constant. When the non-genetic terms are not identifiable, their estimates should not be interpreted. Even in such a case though, the contribution of DGE and SGE ($\sigma_{A_{D}}^{2}$ and $\sigma_{A_{S}}^{2}$), the key quantities we study in our work, should still be correct.

1. Bergsma R, Kanis E, Knol EF, Bijma P. The contribution of social effects to heritable variation in finishing traits of domestic pigs (Sus scrofa). Genetics. 2008;178(3):1559-70.

2. Bijma P. Multilevel selection 4: modeling the relationship of indirect genetic effects and group size. Genetics. 2010;186(3):1029-31.

3. Bijma P. Estimating indirect genetic effects: precision of estimates and optimum designs. Genetics. 2010;186(3):1013-28.

4. Bijma P, Muir WM, Ellen ED, Wolf JB, Van Arendonk JA. Multilevel selection 2: estimating the genetic parameters determining inheritance and response to selection. Genetics. 2007;175(1):289-99.

5. Bijma P, Muir WM, Van Arendonk JA. Multilevel selection 1: quantitative genetics of inheritance and response to selection. Genetics. 2007;175(1):277-88.

6. Canario L, N. Lundeheim and P. Bijma. Pig growth is affected by social genetic effects and social litter effects that depend on group size. Proceedings of the 9th World Congress on Genetics Applied to Livestock Production German Society of Animal Science, Leipzig, Germany. 2010:87.

7. Kang HM, Sul JH, Service SK, Zaitlen NA, Kong S-y, Freimer NB, et al. Variance component model to account for sample structure in genome-wide association studies. Nature genetics. 2010;42(4):348-54.

8. Searle SR. Matrix algebra useful for statistics: Wiley; 1982.

9. Speed D, Balding DJ. Relatedness in the post-genomic era: is it still useful? Nature Reviews Genetics. 2015;16(1):33-44.

10. Legarra A. Comparing estimates of genetic variance across different relationship models. Theoretical population biology. 2016;107:26-30.
