# Supplementary figures and images for: Genetic Variation in the Social Environment Contributes to Health and Disease
Source: PLoS Genet. 2017 Jan 25;13(1):e1006498. doi: 10.1371/journal.pgen.1006498 (PMC5266220; doi:10.1371/journal.pgen.1006498)

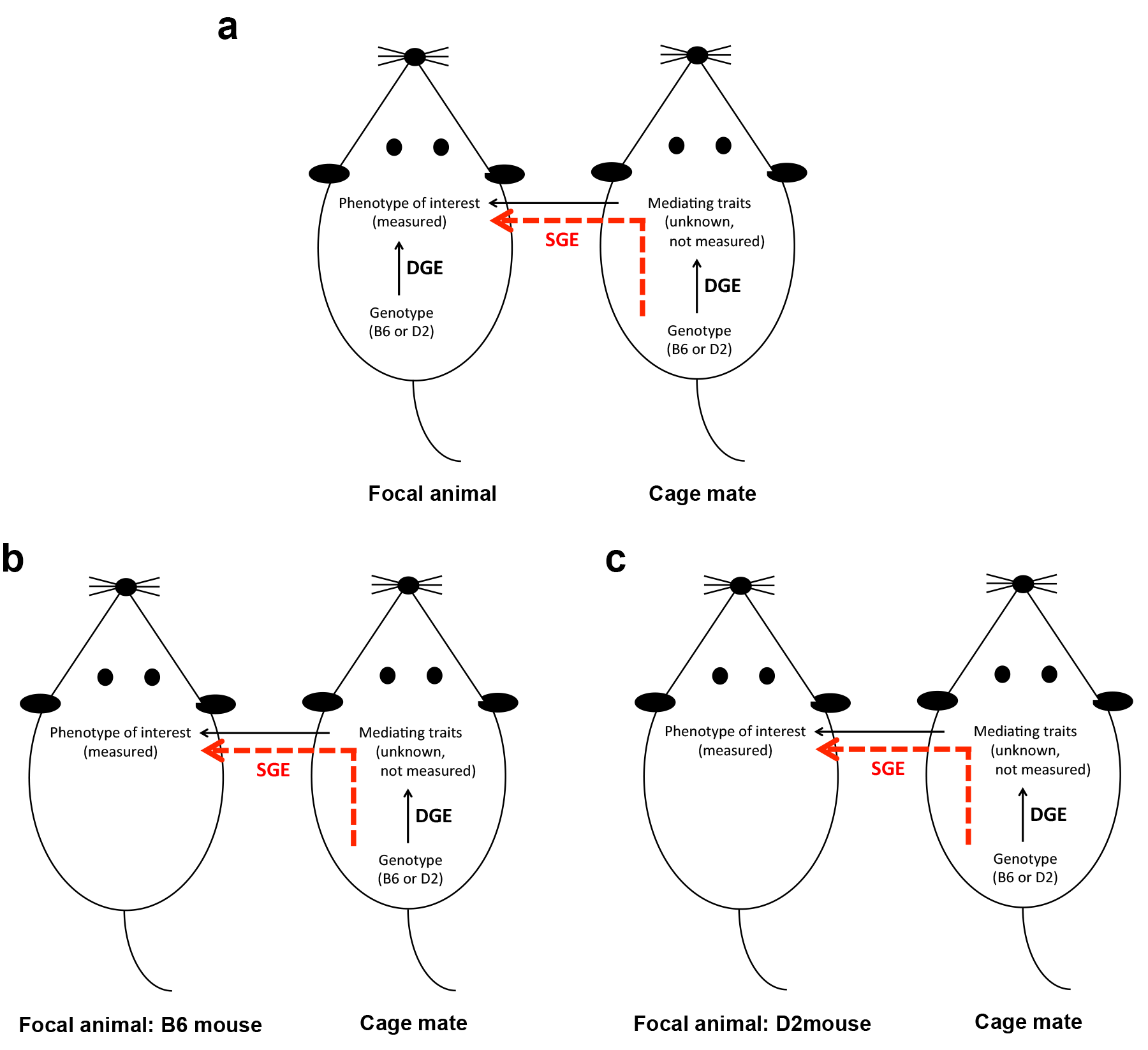

Supplement: S1 Fig — (A) When jointly analyzing B6 and D2 focal mice (for S2 Fig and columns “all mice” of S2 Table), DGE, SGE and their interaction can be detected. All other results were obtained by considering B6 focal mice (B) and D2 focal mice (C) separately. The only genetic parameter then is the strain of the cage mate. Environmental effects are not shown. (TIF) [file pgen.1006498.s002.tif]

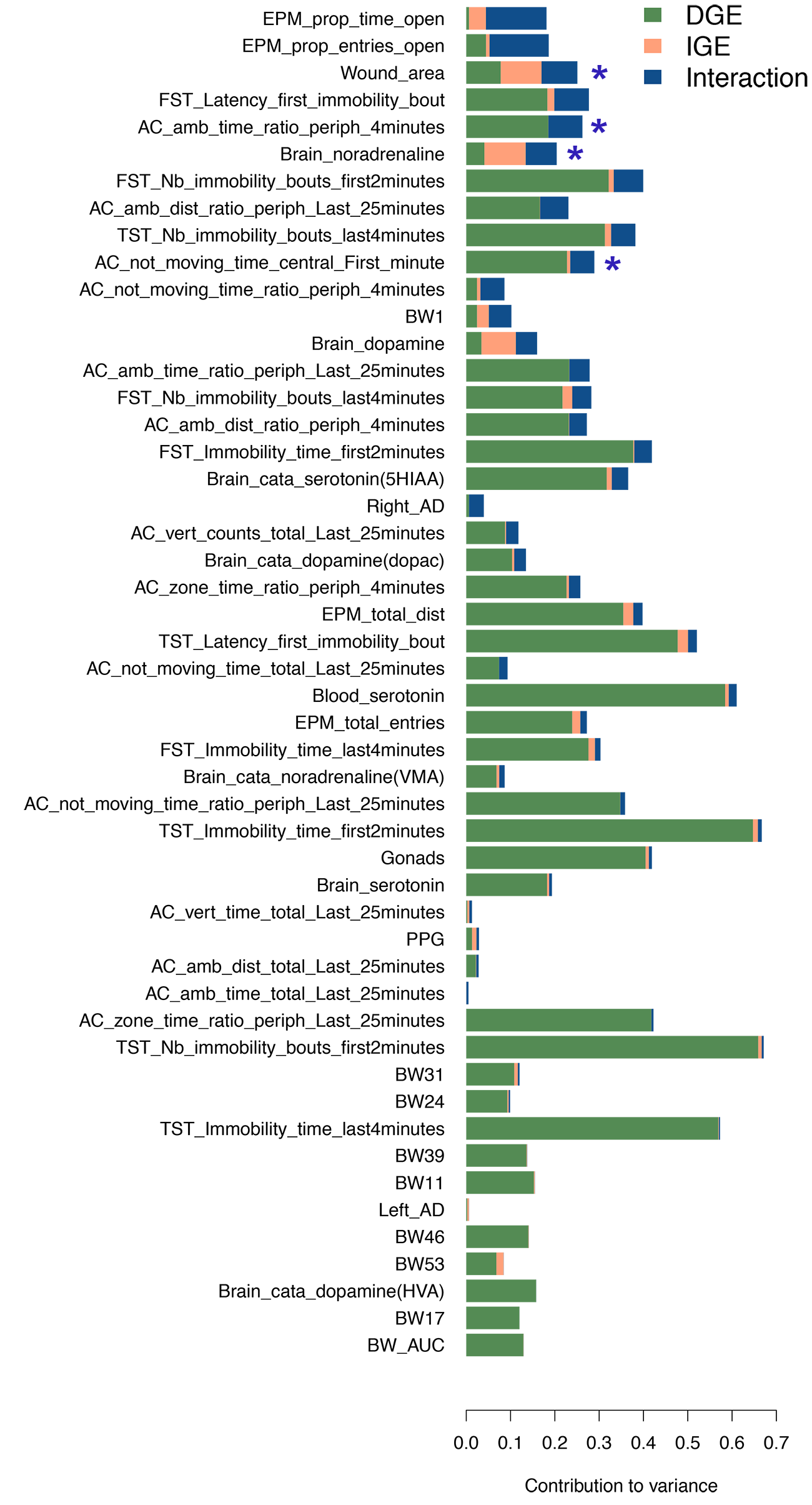

Supplement: S2 Fig — The contribution to phenotypic variance of DGE, SGE, and their interaction is shown. All organismal phenotypes are shown (see S1 Table for a description of each measure). They are plotted in order of decreasing contribution of the interaction component. Blue stars indicate phenotypes for which a model with interaction was selected based on the Akaike information criterion (AIC). (TIF) [file pgen.1006498.s003.tif]
